# Restorative Strategies for Bilateral Mutilated Hands in a Secondary Care Level: A Report of a Case in Mexico

**DOI:** 10.7759/cureus.56036

**Published:** 2024-03-12

**Authors:** Kenneth Aleman Paredes, Julio C Selaya Rojas, Carina L Nolasco Mendoza, Alberto Acosta Ramirez, Mauricio Montelongo Quevedo, Jose R Flores Valdés

**Affiliations:** 1 Surgery, Hospital General Regional No 220 "José Vicente Villada", Toluca, MEX; 2 Plastic and Reconstructive Surgery, Hospital General Regional No 220 "José Vicente Villada", Toluca, MEX; 3 Surgery, Hospital General Regional No 220 "Jose Vicente Villada", Toluca, MEX; 4 General Medicine, Universidad Autónoma de Guadalajara, Guadalajara, MEX; 5 General Medicine, Universidad Autonoma de Guadalajara, Guadalajara, MEX

**Keywords:** hand and cosmetic surgeon, general trauma surgery, hand surgeon, complex trauma, plastic and reconstructive surgery

## Abstract

This case report aims to delineate the challenges and management strategies for a patient with bilateral mutilated hands within a secondary care level in Mexico, contributing to medical literature and potentially guiding future patient care. Mutilated hands represent a significant surgical and rehabilitative challenge due to the profound structural damage they cause, leading to considerable functional impairment and psychological distress. The complexity of these injuries necessitates a multidisciplinary approach, particularly in resource-constrained settings. We present a case of a 45-year-old male with no prior significant medical history who sustained bilateral mutilated hands from an industrial accident involving hot rollers. The patient underwent extensive surgical reconstruction and postoperative care, facing complications such as skin graft integration issues and infections, which required a multidisciplinary treatment approach.

## Introduction

Mutilated hand injuries, marked by profound structural damage, stand as a pinnacle of complexity and severity within the realm of hand surgery. These injuries not only cause significant functional limitations but also lead to intense psychological suffering. Particularly in settings where industrial and agricultural mishaps are prevalent, the impact of hand injuries on trauma care is profound and far-reaching. This underscores the critical need for specialized care and attention in managing such cases [1].

The definition of a mutilating hand injury is multifaceted. Deriving from the Latin term for "to cut or trim away," the term "mutilating" describes injuries that lead to significant tissue loss, compromised function, and diminished esthetic value of the hand. A pivotal concept in this context is the "acceptable hand," which is essentially a hand with three fingers of near-normal length, satisfactory motion in the proximal interphalangeal joints, adequate tactile sensitivity, and an operational thumb. Injuries that prevent the hand from meeting these criteria are thus categorized as mutilating [2].

The American Medical Association's guidelines for evaluating permanent impairment provide an insightful framework, illustrating the unique functional contributions of each digit to the hand, upper extremity, and overall body function. Highlighting the thumb's critical role, a loss of this digit is equated to a 40% reduction in hand functionality and a 25% diminution in total body function. Though other fingers are not valued as highly, each plays a vital role in hand functionality, from precision grips facilitated by the radial digits to the strong grasp supported by the ulnar digits [3].

Hand mutilation encompasses injuries causing extensive damage to multiple tissues simultaneously. The variability in these injuries stems from the countless possibilities in terms of severity, range, and number of tissues affected, making standardized treatment approaches impractical. Nonetheless, it is possible to define fundamental principles for their surgical management. Surgeons handling such cases need to thoroughly understand these principles to guide their decision-making and thus influence the recovery outcomes. Developing a detailed treatment strategy focused on specific objectives is key to achieving swift functional and psychological rehabilitation. However, the journey can be marred by setbacks, including the need for multiple surgeries and potential failures, which might lead to less favorable results. Today's ability to restore functionality to an injured hand is the fruit of advancements across various medical disciplines [4].

This case report documents a rare and formidable challenge: the treatment of bilateral mutilated hands at a secondary care level in Mexico. This case's complexity, exacerbated by limited resources, stands as a significant point of interest. The objective of sharing this experience is to enrich the medical literature and enhance future patient care strategies.

The insights gained from managing such a complex case offer valuable lessons for the treatment of mutilating hand injuries, especially in similar resource-constrained environments. These experiences could assist in future treatment decisions and strategies, potentially setting new precedents for handling such intricate injuries across diverse clinical settings.

## Case presentation

**Figure 1:**
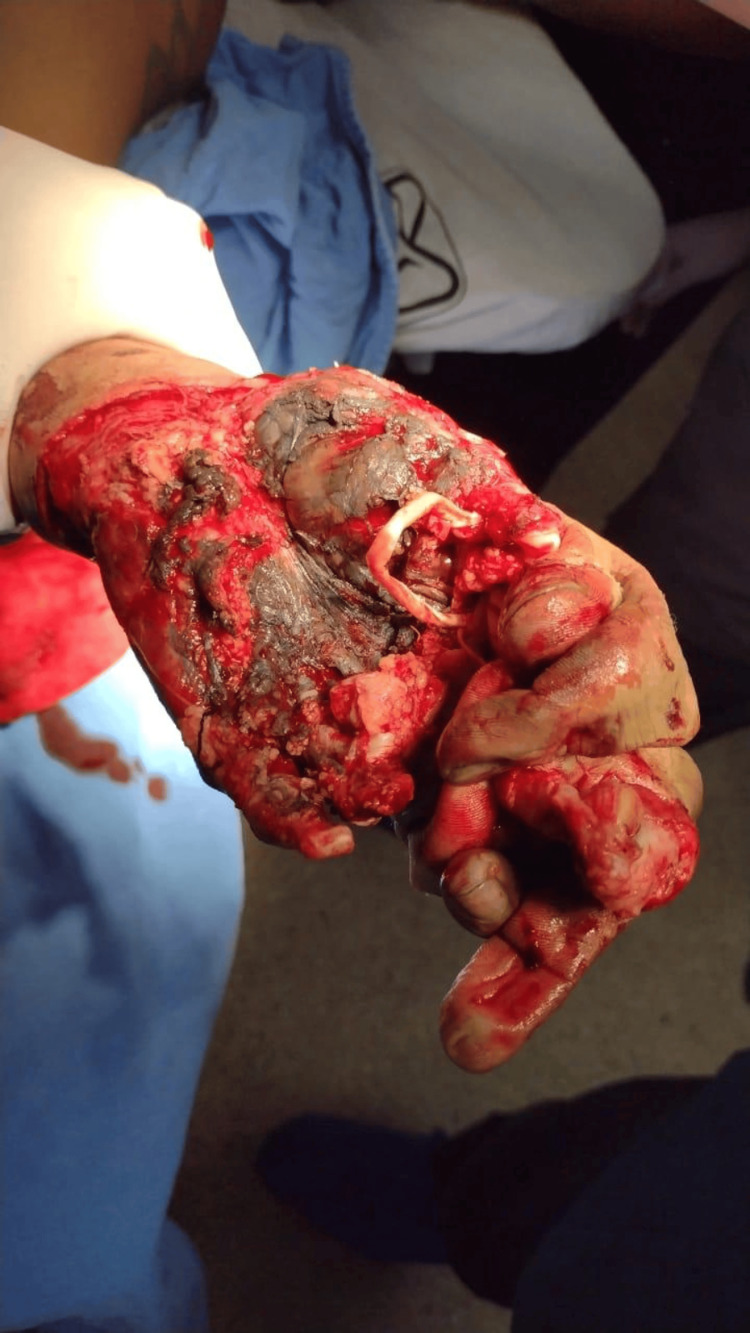
Initial presentation of left hand with a severe avulsion hand trauma.

**Figure 2:**
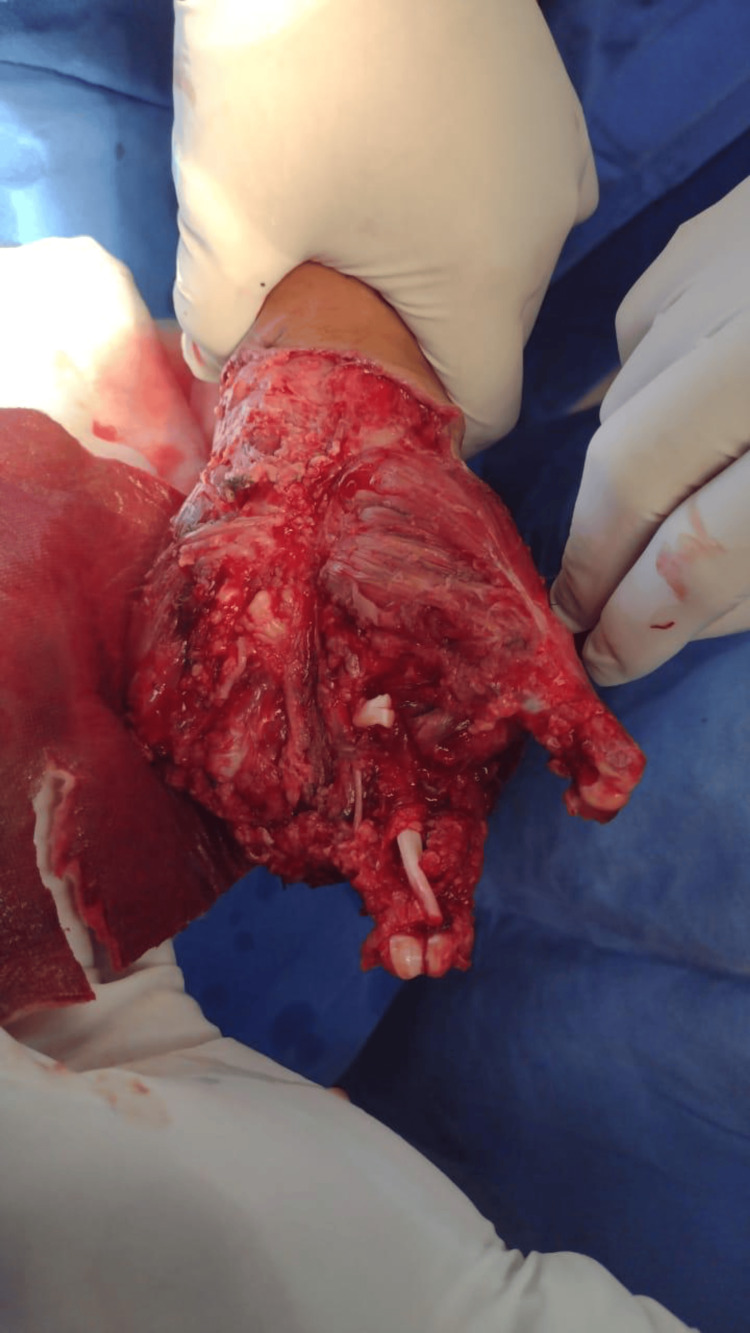
Note the complex nature of the trauma with extensive tissue damage and visible bone fragments.

A 45-year-old male, with no significant medical history before the recent accident, was brought to the emergency room by coworkers. He had sustained injuries while cleaning hot rollers; while attempting to reach an object, both of his hands became trapped in the rollers, resulting in a crush and avulsion trauma (Figures 1, 2).

In the emergency room, the patient reported acute and severe pain along with soft-tissue trauma and active bleeding. Upon physical examination, the patient was oriented in time and place, with a Glasgow score of 15. The upper limbs showed mobility arches at the shoulder and elbow levels without limitation. Severe soft-tissue trauma was observed at the hands, with loss of skin cover, bone exposure, and loss of distal phalanges in the first, middle, and distal fingers. Additionally, there was loss of muscle, tendons, and vascular injury. The rest of the physical examination was not relevant. 

The diagnosis given by the Emergency Room department was bilateral mutilated hands.

Before admission, laboratory tests were performed (Table 1).

**Table 1:** Patient lab values taken prior to admission.

Parameter	Value	Reference Values	Unit
Glucose	112.4	70-110	mg/dL
Creatine Phosphokinase Total	299.3	22-198	U/L
Creatine Phosphokinase-MB	51.1	0-25	U/L
Hemoglobin	14.9	13.5-17.5	g/dL
Hematocrit	43.7	41-53	%
Leukocytes	4.8	4.5-11	x10^9^/L
Platelets	207	150-400	x10^9^/L
PT (Prothrombin Time)	11.4	11 to 15	Seconds
INR (International Normalized Ratio)	1.04	0.8-1.2	-
aPTT (Activated Partial Thromboplastin Time)	27.3	25 to 40	Seconds

**Figure 3:**
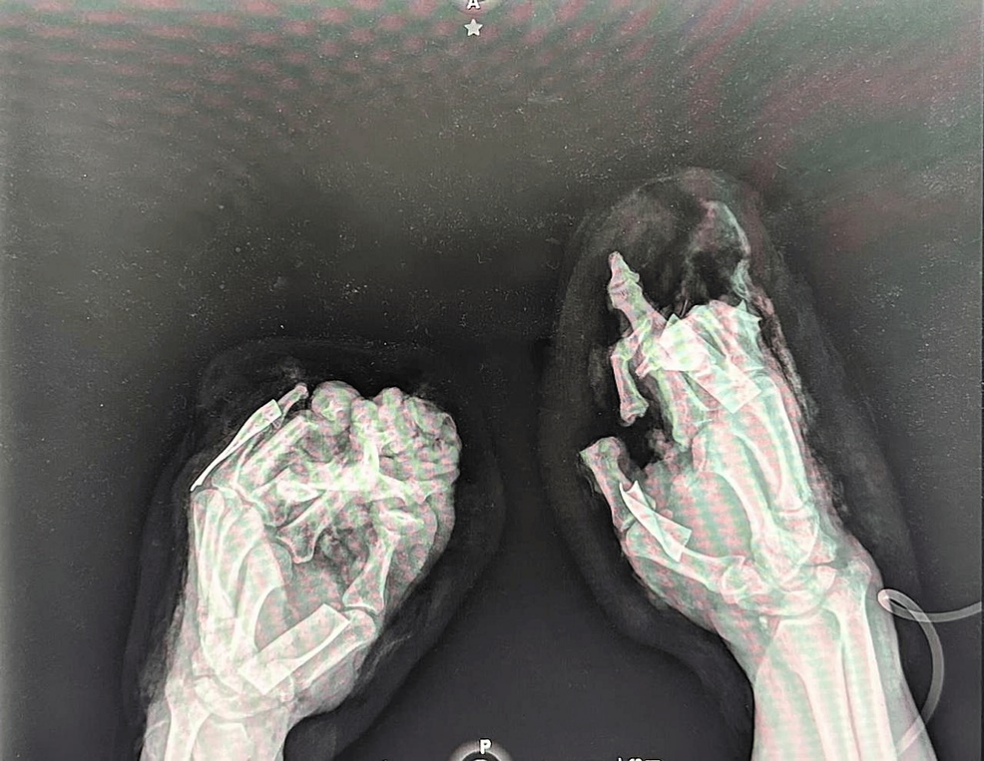
Preoperative X-ray, oblique projection of both hands. The radiographic image demonstrates the traumatic amputations of the left hand involving loss of proximal, middle, and distal phalanges of the third, fourth, and fifth fingers, and the distal phalanx of the first finger.

**Figure 4:**
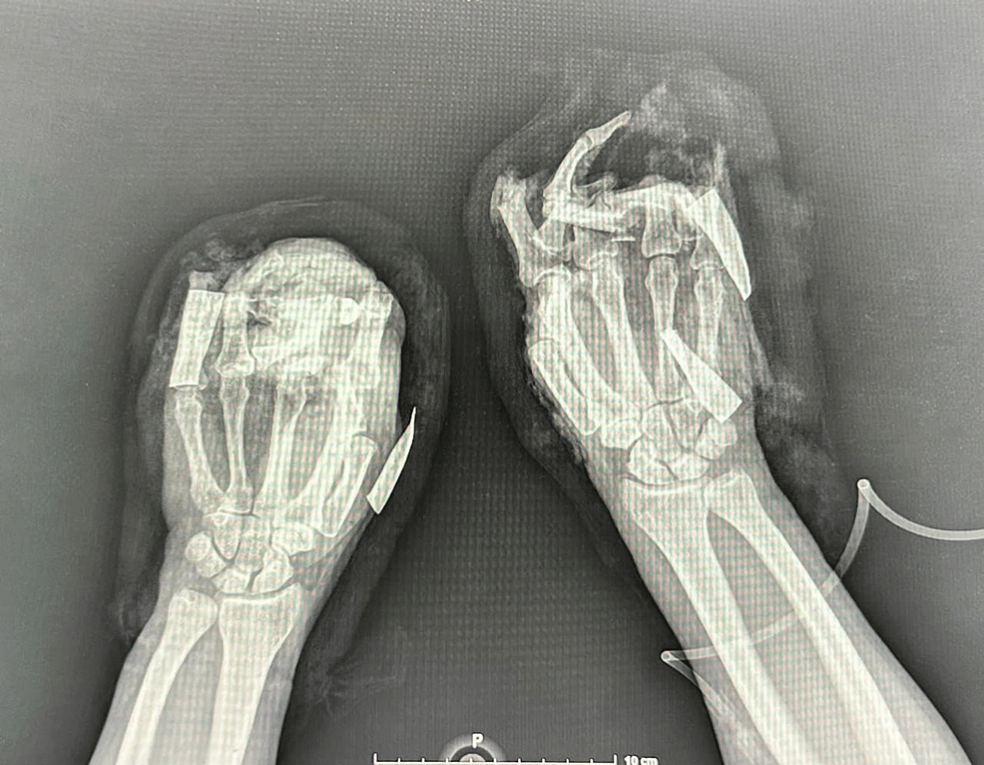
Anteroposterior projection of both hands. This radiographic image reveals the extensive injuries sustained to the right hand, including the loss of middle and distal phalanges of the second finger, and multiple fractures and skeletal disruptions consistent with a crushing mechanism of injury.

X-rays were conducted prior to admission (Figures 3, 4).

**Figure 5:**
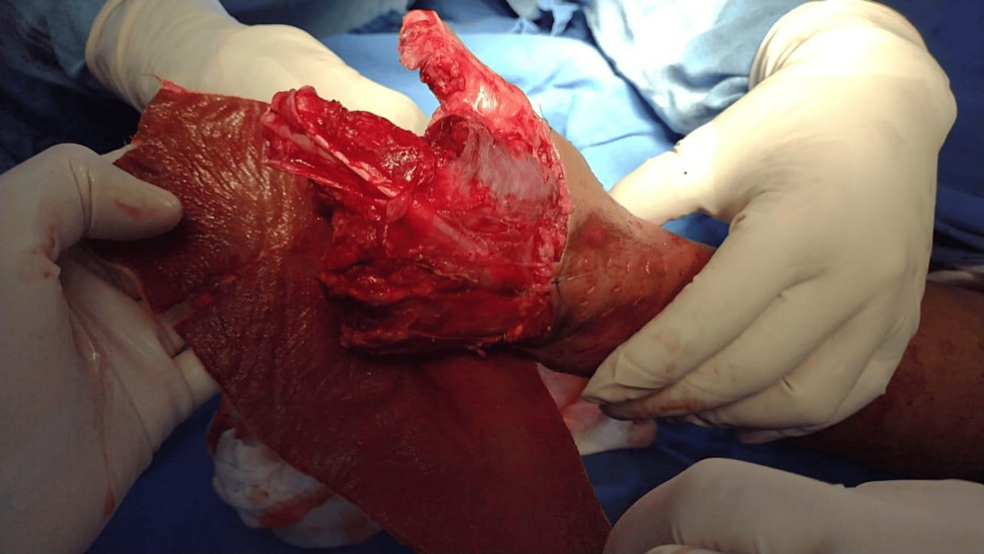
Intraoperative view before skin grafting.

Upon the patient's admission, the hand surgery team verified the diagnosis of bilateral mutilating hand injury. A strategic approach was formulated, prioritizing functional reconstruction and esthetic restoration. The intricate procedure entailed harvesting thick partial skin grafts from the patient's left hand's residual hanging tissue. The patient's own skin cells, harvested from the remaining skin after a degloving injury, were carefully used to graft onto the right hand, ensuring closure of the wound with autologous tissue, optimizing the chances of graft acceptance and functional recovery. To protect the vulnerable postoperative tissue and promote healing, a silver-impregnated dressing was applied (Figure 5).

**Figure 6:**
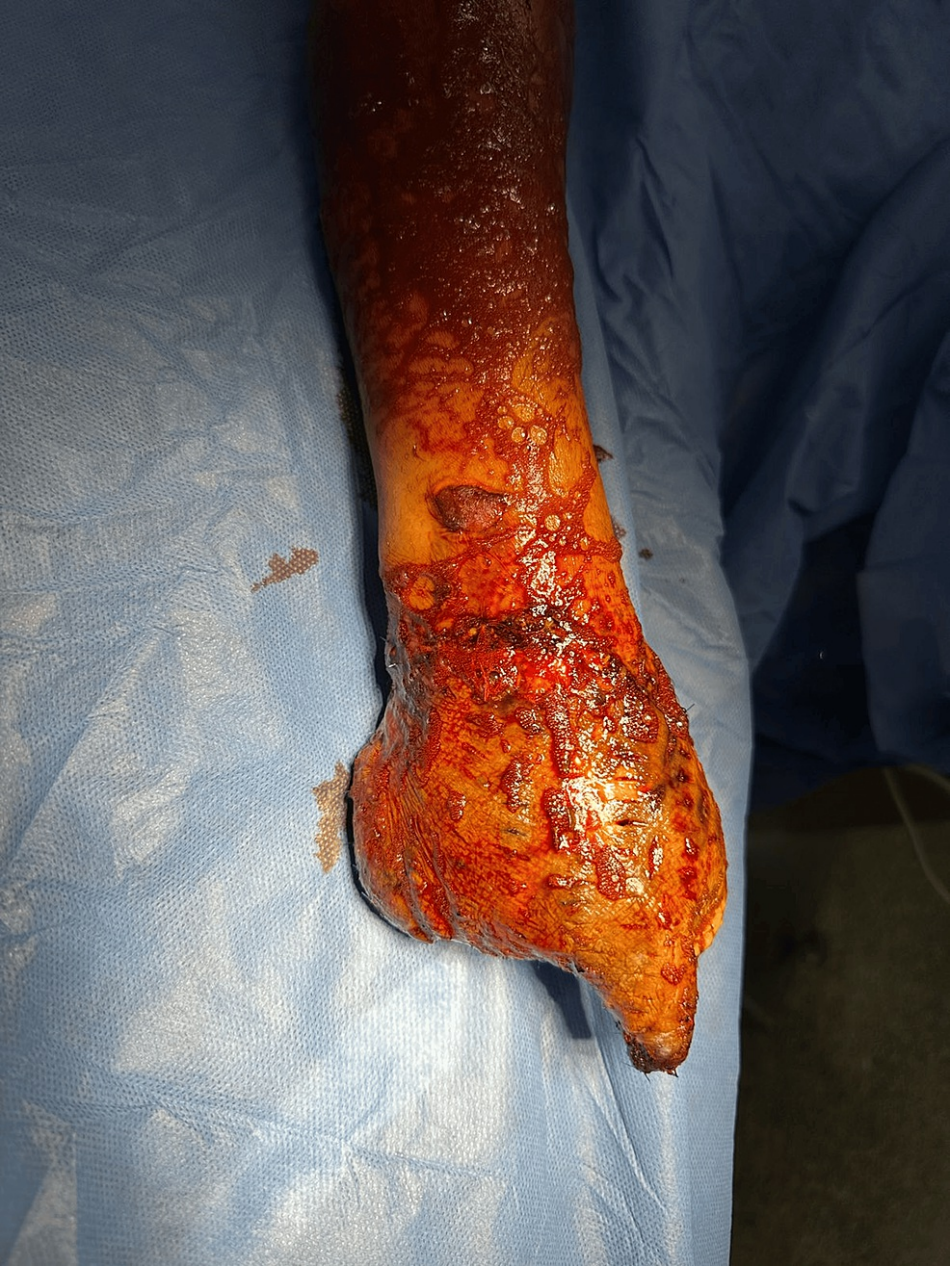
Patient’s right hand following debridement. The image shows extensive tissue damage with visible bone structures, indicative of the severity of the crush and avulsion injury. The preparation of the wound bed for grafting is critical to facilitate successful graft take and optimal functional recovery.

**Figure 7:**
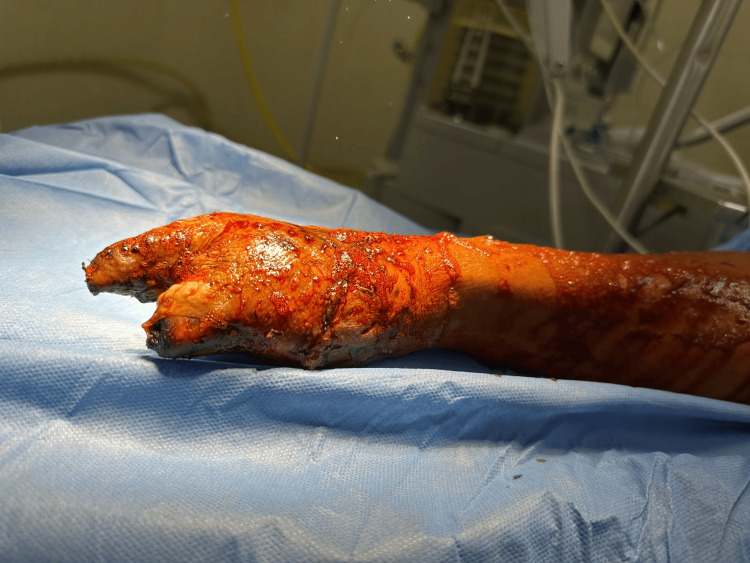
Initial postoperative condition of the skin graft on the right upper extremity following debridement.

After surgery, the patient faced complications such as issues with skin graft integration and multiple antiseptic wound care that proved insufficient against recurrent fungal and gram-negative infections. Consequently, multidisciplinary specialties were involved, including the Department of Immunology and Infectious Diseases, which treated the patient with broad-spectrum antibiotics and antifungal medications (Figures 6, 7).

**Figure 8:**
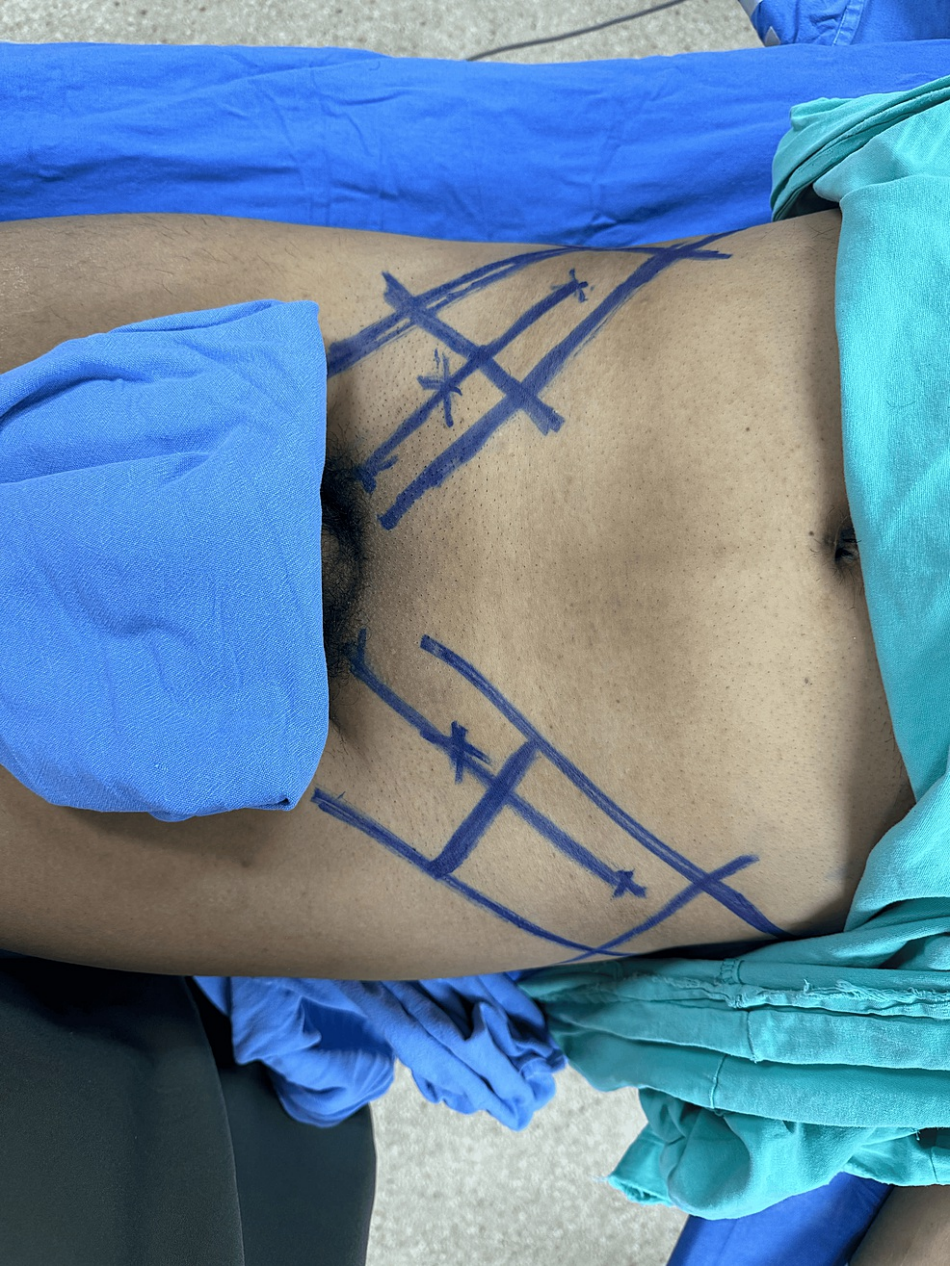
This image illustrates the carefully planned and marked donor site on the patient's body, designated for harvesting skin grafts. The markings indicate the precise area and dimensions of the skin to be excised for grafting purposes. Such planning is crucial to ensure that the size and shape of the harvested skin adequately cover the recipient site, which in this case is intended for a McGregor flap procedure.

**Figure 9:**
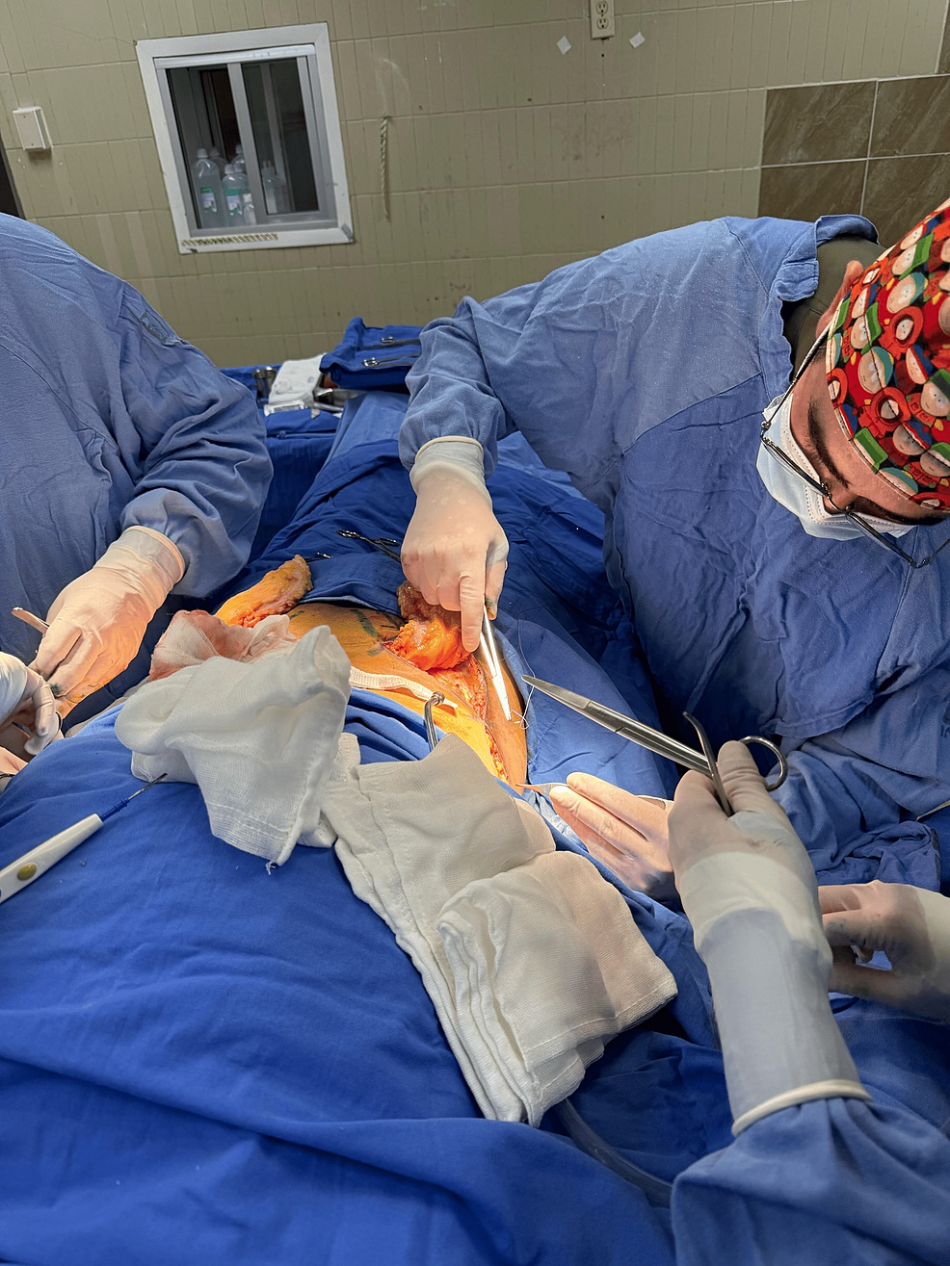
Dr Aleman during the intraoperative preparation for the McGregor flap procedure. The image displays the meticulous surgical technique employed in preparing the recipient site for a McGregor flap on a burn patient.

**Figure 10:**
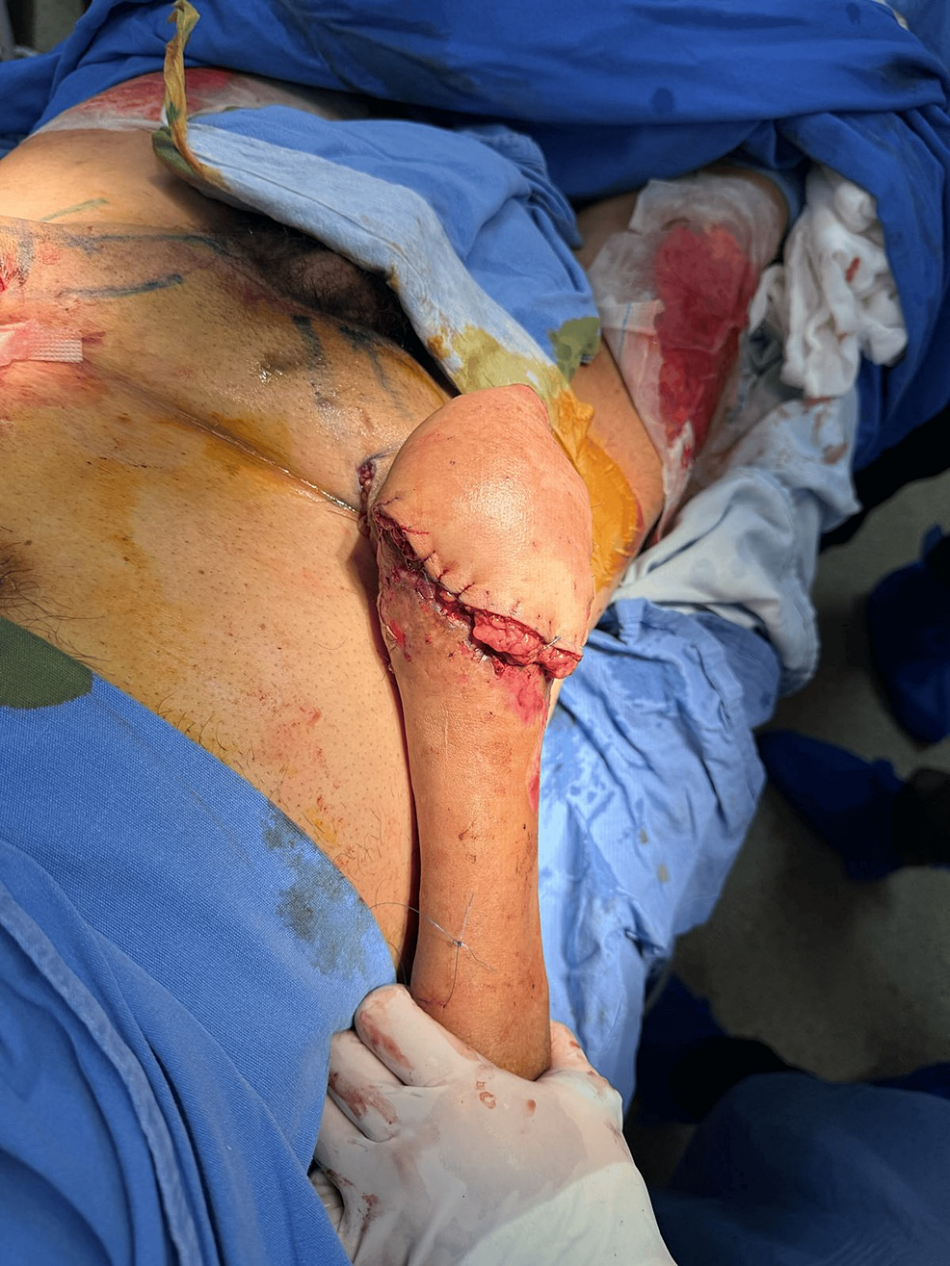
Postoperative view of McGregor flap and skin graft placement. This image showcases the immediate postoperative results following the placement of a McGregor flap and skin graft. The flap is meticulously sutured in place, demonstrating the intricate technique required for such procedures. The skin graft, harvested from the predetermined donor site, has been secured over the area, initiating the process of integration and healing. This step is critical in reconstructive surgery, aiming to restore the function and appearance of the area affected by trauma or excision.

Hospitalization was prolonged due to persistent and recurrent infections until cultures returned negative. After 40 days, once the cultures were confirmed negative, the Plastic and Reconstructive Surgery department decided to proceed with the next surgery intervention. This involved creating a MacGregor inguinal flap and placing a thick partial skin graft, which was harvested from the anterior face of both thighs (Figures 8-10).

**Figure 11:**
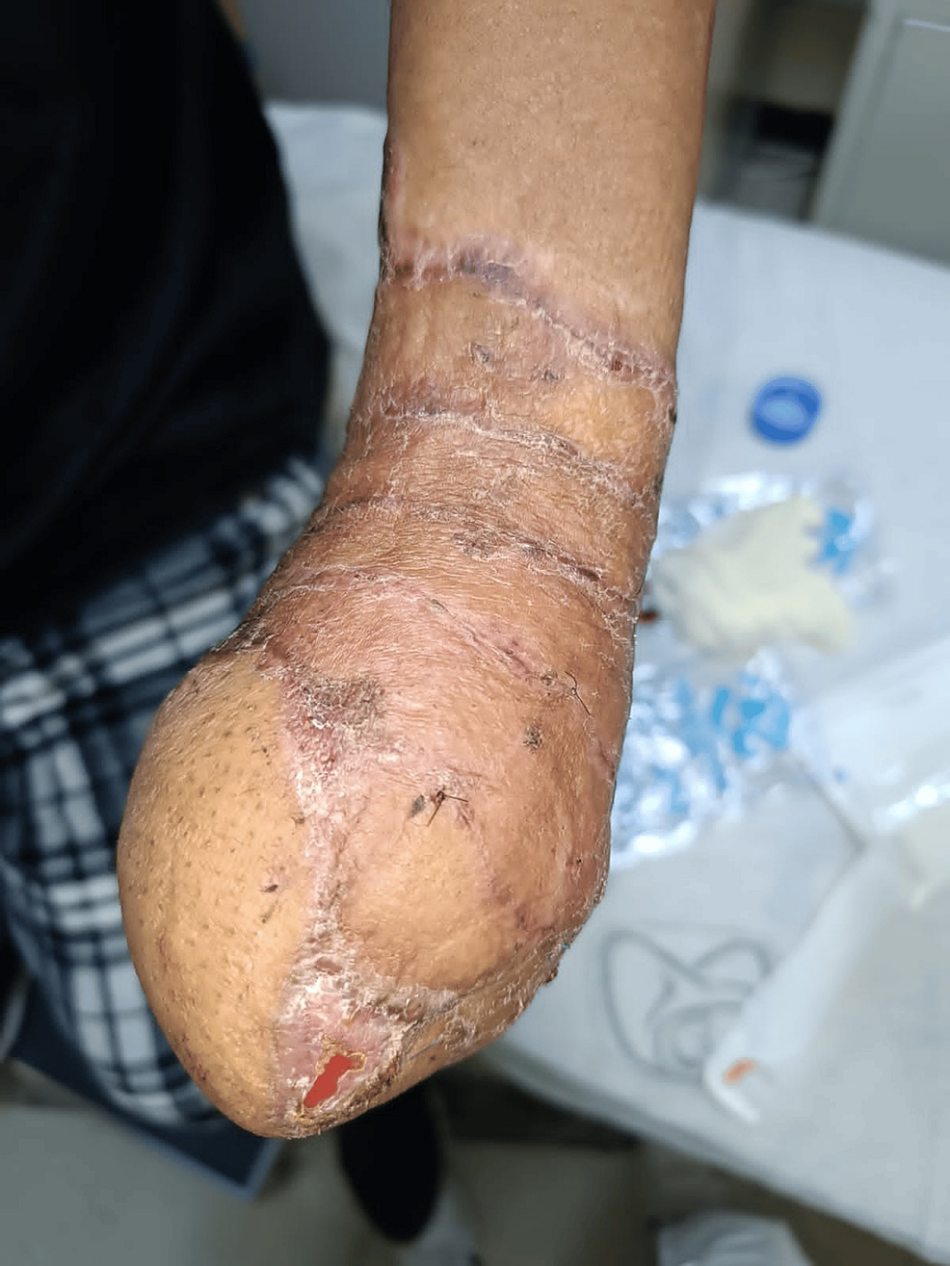
Healed surgical site after McGregor flap release.

**Figure 12:**
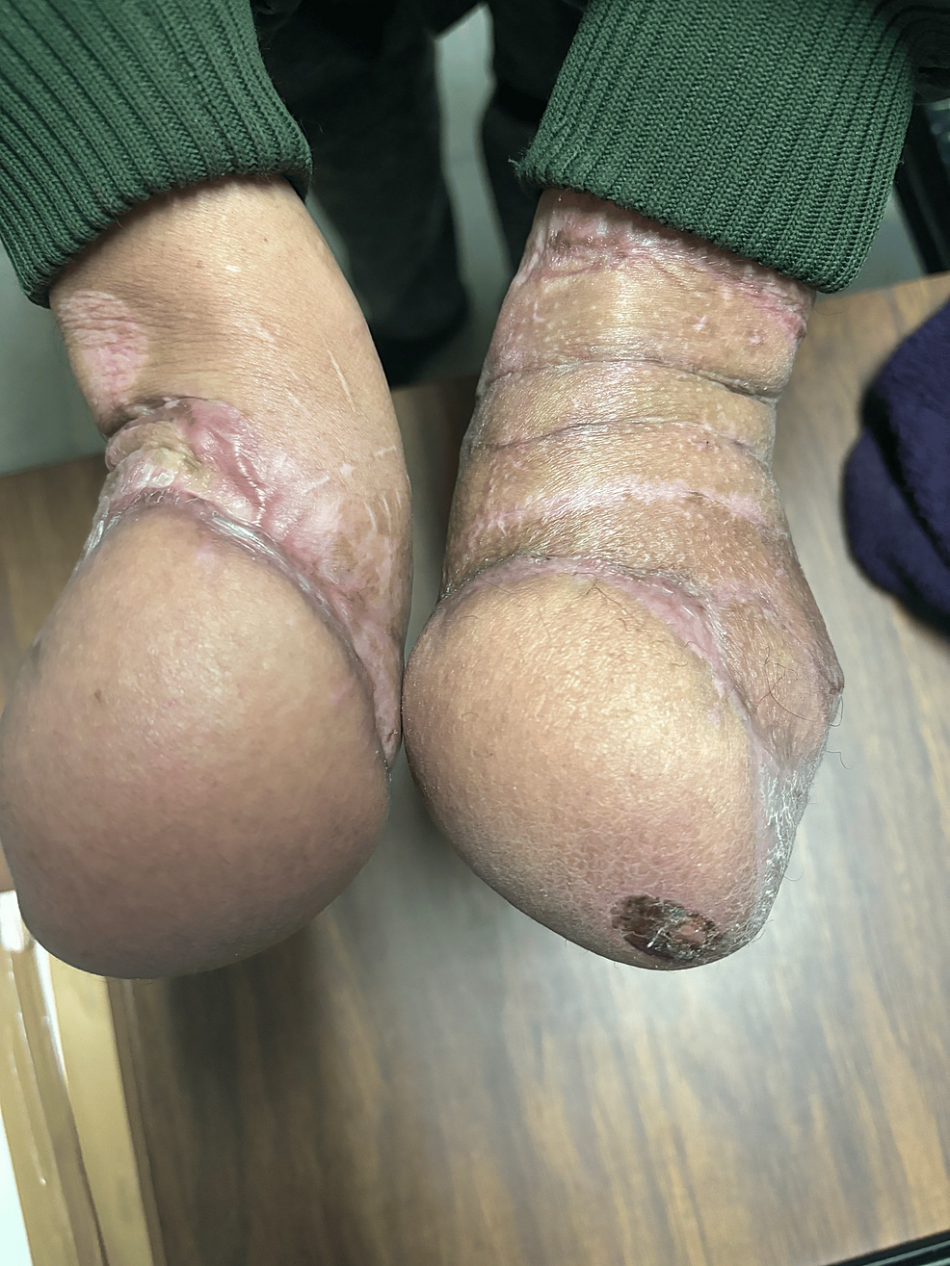
This image captures the patient’s healed forearm after three months of flap release, undergoing twice-weekly outpatient follow-ups for wound care management.

The patient underwent outpatient follow-ups twice a week for wound care management and was under surveillance for three weeks. After four weeks of surveillance, the patient underwent flap release due to good integration and no further complications, leading to the decision for discharge (Figures 11, 12).

In three months’ time, in May 2024, we plan to re-evaluate the patient's condition to assess the feasibility of the digitalization of both hands. The next intervention will be to restore a functional index and a functional thumb. Our multidisciplinary team will focus on the patient's functional needs and quality-of-life improvements, aiming to enhance dexterity and hand utility. The decision to proceed with digitalization will be contingent on the patient achieving optimal conditions for the best possible surgical outcome.

## Discussion

The management of mutilated hands requires meticulous and comprehensive care due to the profound functional and psychological impacts these injuries impose. Our case report presents a patient with bilateral mutilated hands treated at a secondary-level care facility in Mexico, underscoring the challenges and the resourcefulness needed in settings with limited resources.

Mutilating hand injuries entail significant tissue loss and functional compromise, necessitating a classification for a clear approach to treatment. The Reid classification system, which categorizes such injuries into seven types, provides a framework for understanding and strategizing the management of these complex injuries. However, another classification system exists [5], highlighting the need to standardize one.

The case at hand involved a 45-year-old male who experienced a catastrophic industrial accident leading to bilateral hand mutilation. This case exemplifies not only the severity of the injury but also the critical role that timely and appropriate medical intervention plays in the management of such cases. The initial care provided in the emergency department was crucial for stabilizing the patient and preventing further morbidity, emphasizing the importance of a well-coordinated trauma system, even in secondary care settings.

Laboratory tests and radiographic imaging played a pivotal role in the initial assessment and planning of surgical intervention. The loss of phalanges and extensive tissue damage presented challenges that were magnified by the bilateral nature of the injuries and the complications that ensued postoperatively, including difficulties with graft integration and infections.

The complications experienced by the patient highlight the necessity of a multidisciplinary approach in the management of mutilating hand injuries. The involvement of Infectology specialists, who administered broad-spectrum antibiotics and antifungal medications, was integral to overcoming the persistent infections that the patient suffered postoperatively. This aspect of care is particularly critical in secondary care settings where the prevalence of antibiotic-resistant organisms may be higher, and resources for advanced care may be scarcer, as reported by Kumta et al. [6].

Despite these challenges, the ingenuity of the surgical team and the adaptability of treatment strategies, such as the use of the MacGregor inguinal flap, demonstrate the potential for successful outcomes even in less-resourced settings. The successful integration of the skin grafts and the eventual release of the flap after intensive outpatient follow-up care culminated in a positive outcome for a seemingly dire situation.

The patient’s recovery path highlights the critical role of wound debridement in the initial stages to ensure all debris is thoroughly removed. Vigilant postoperative care, including rigorous infection control, is essential for subsequent wound optimization. The prolonged hospitalization due to recurrent infections illustrates the dynamic nature of treating mutilated hand injuries and the need for persistence in care strategies. Furthermore, the case demonstrates the feasibility of achieving satisfactory outcomes in the management of complex hand injuries in a second-level care setting with a comprehensive, multidisciplinary approach.

This case contributes to the existing body of literature by providing insights into the management of complex hand injuries in a resource-limited setting, potentially guiding future treatment strategies and decision-making processes. It highlights the critical need for capacity building in secondary care facilities in regions where industrial and agricultural accidents are prevalent, and the importance of early surgical intervention, as in Neumeister and Brown [7]. This report also underscores the importance of the likelihood of a second procedure being needed for a useful hand, aligning with the importance as described in Russell et al. [8].

Finally, this report underscores the broader implications for the management of mutilated hands, advocating for the development of protocols and the integration of multidisciplinary teams to optimize patient outcomes in similar clinical scenarios. Future research should focus on the long-term functional and psychological outcomes of patients with mutilated hands, exploring the efficacy of various reconstructive strategies and rehabilitation protocols in enhancing the quality of life after injury. In this case, the McGregor flaps were used, but there are cases with more complexity, such as in Wei et al. [9].

## Conclusions

This case report delineates the profound challenges and complexities encountered in managing bilateral mutilated hands at a secondary care facility in Mexico. The severe functional impairment and psychological trauma associated with such catastrophic injuries demand immediate, comprehensive surgical intervention and vigilant postoperative care. Highlighting the urgency of early debridement and reconstructive procedures, this case demonstrates their significance in averting life-threatening complications and laying the groundwork for potential functional rehabilitation.

Our experience underscores the necessity of an adaptable, resourceful approach amidst resource constraints. Although such injuries are typically addressed in tertiary care centers equipped with extensive resources, this report showcases the capability of a secondary care facility to deliver critical, limb-saving treatment. The success achieved in this challenging setting can be attributed to the prompt action by the surgical team and the cohesive efforts of a multidisciplinary team, tackling various care continuum challenges.

Furthermore, this case accentuates the importance of multidisciplinary collaboration in meeting the complex needs of patients with mutilated hands. The invaluable contributions from Infectology, Plastic and Reconstructive Surgery, and other specialties were crucial in managing persistent infections and facilitating the integration of skin grafts and the McGregor flap.

This report enriches the medical literature by demonstrating that favorable outcomes are attainable in complex hand injury management within a secondary care context. It highlights the resilience and creativity needed in resource-limited settings and advocates for the development of customized protocols that maximize available resources.

The insights from this case inform future care strategies and underline the importance of enhancing capacity in secondary care facilities, especially in areas prone to industrial and agricultural accidents. Future research should focus on the long-term functional and psychological rehabilitation of patients with mutilated hands, evaluating the efficacy of different surgical and rehabilitative techniques in enhancing life quality.

In summary, managing bilateral mutilated hands in a secondary care setting, despite its challenges, is achievable. Through the dedicated efforts of a multidisciplinary team and a strategic, patient-centric approach to care, navigating the complexities of such cases and significantly improving patient outcomes is possible. This case report stands as a testament to the human spirit's resilience and medical professionals' commitment to restoring hope and functionality against daunting odds.
